# Supplementary material for: Efficient excitation and control of integrated photonic circuits with virtual critical coupling
Source: Nat Commun. 2024 Mar 28;15:2741. doi: 10.1038/s41467-024-46908-2 (PMC10978855; doi:10.1038/s41467-024-46908-2)
Supplement: Supplementary file 1 — Supplementary Information [file 41467_2024_46908_MOESM1_ESM.pdf]

Efficient excitation and control of integrated photonic circuits with virtual critical coupling

Jakob Hinney[1,*], Seunghwi Kim[2,*], Graydon J. K. Flatt[3], Ipshita Datta[1], Andrea Alù[2,4,†], Michal Lipson[1,3,†]

[1]*Department of Electrical Engineering, Columbia University, New York, NY 10027, USA*

[2]*Photonics Initiative, Advanced Science Research Center, City University of New York, New York, NY 10031, USA*

[3]*Department of Applied Physics and Applied Mathematics, Columbia University, New York, NY 10027, USA*

[4]*Physics Program, Graduate Center, City University of New York, New York, NY 10016, USA*

[†]*Corresponding authors: ml3745@columbia.edu, aalu@gc.cuny.edu*

[*]These authors contributed equally to this work

I. Virtual critical coupling

In this section, we thoroughly investigate the temporal evolution of the transmission coefficient under a complex frequency excitation. Our experimental setup, as discussed in the main text, can be modeled using coupled mode theory [1]. The equation of motion for the intracavity field *a* is written as

$$\dot{a} = -\left(\kappa + i\omega_0\right)a + \sqrt{2\kappa_{ex}}\, a_{in.0} e^{-i\omega_L t}, \tag{S.1}$$

where $\kappa = \kappa_i + \kappa_{ex}$ is the total loss rate consisting of the intrinsic loss rate κ_i and external coupling rate κ_{ex} to a bus waveguide. ω_0 is the resonant frequency, ω_L is the laser frequency, and $a_{in,0}$ is the input amplitude. Here, the laser frequency has a complex frequency $\omega_L = \omega_{re} + i\omega_{im}$, and we change the frame of Eq. (S.1) to the real part of ω_L , i.e., $a^{-i\omega_{re}t} \to a$, yielding

$$\begin{aligned} \dot{a} &= -(\kappa + i\Delta)a + \sqrt{2\kappa_{ex}}a_{in}, \\ a_{out} &= a_{in} - \sqrt{2\kappa_{ex}}a, \end{aligned} \tag{S.2}$$

where a_{out} is the output field and $\Delta = \omega_0 - \omega_{re}$ is the detuning of the pump laser frequency. Here we introduce the input field, including the exponential growth term, i.e., $a_{in}(t) \equiv a_{in,0}e^{\omega_{im}t}$. The second term in Eq. (S.2) represents the output field measured at the end of the waveguide formulated by the input-output theory [2, 3]. To obtain the analytical expression for the transmission under complex frequency excitation in Eq. (1) of the main text, we focus on the case where $a_{in,0}$ is constant.

We can solve Eq. (S.2) to obtain a(t) with a given initial condition that a(0) = 0, and the solution is given by

$$\begin{aligned} a(t) &= e^{-(\kappa+i\Delta+\omega_{im})t}\left[\sqrt{2\kappa_{ex}}a_{in}\int e^{(\kappa+i\Delta+\omega_{im})\tau}d\tau + c\right] \\ &= \frac{\sqrt{2\kappa_{ex}}a_{in}}{\kappa + i\Delta + \omega_{im}}\left[1 - e^{-(\kappa+i\Delta+\omega_{im})t}\right]. \end{aligned} \tag{S.3}$$

Considering the input-output formula in Eq. (S.2), we obtain the transmission coefficient:

$$\tilde{T}(t) \equiv \frac{a_{out}}{a_{in}} = 1 - \frac{2\kappa_{ex}}{\kappa + i\Delta + \omega_{im}}\left[1 - e^{-(\kappa+i\Delta+\omega_{im})t}\right] = \tilde{T}_{qs} + \tilde{T}_{ts}(t). \tag{S.4}$$

As discussed in the main paper, the temporal transmission coefficient in Eq. (S.4) consists of the quasi-steady state $\tilde{T}_{qs} \equiv \frac{\kappa_i - \kappa_{ex} + i\Delta + \omega_{im}}{\kappa_i + \kappa_{ex} + i\Delta + \omega_{im}}$ and the transient state $\tilde{T}_{ts}(t) \equiv \frac{2\kappa_{ex}}{\kappa + i\Delta + \omega_{im}} e^{-(\kappa + i\Delta + \omega_{im})t}$. Here, only the transient state of the transmission changes with time, while the quasi-steady state does not change in time, i.e., it evolves in a quasi-steady state regime oscillating at the same complex frequency as the excitation. For high-Q systems, the transmission coefficient $\tilde{T}(t)$ converges to the quasi-steady state of the transmission $\tilde{T}_{qs}$ at zero detuning $\Delta = 0$ after a few cycles, resulting in zero transmission under the condition $\omega_{im} = \kappa_{ex} - \kappa_i$, as explained in the main text.

In practice, however, an exponentially-growing input pulse $a_{in} \equiv a_{in,0} e^{\omega_{im} t}$ suddenly starts from a nonzero value at t = 0 due to the nature of the exponential function; therefore, we should apply smooth filters to the pulse so it starts at zero. For instance, we can consider the modified input instead [4]

$$\dot{a} = -(\kappa + i\Delta) a + \sqrt{2\kappa_{ex}} \frac{t}{t + T_0} a_{in}. \tag{S.5}$$

Here, T_0 is the time constant for the filter. In this case, the solution of Eq. (S.5) is given by:

$$\begin{aligned} a(t) = &\frac{\sqrt{2\kappa_{ex}} a_{in}}{\kappa + i\Delta + \omega_{im}} \left[1 - e^{-(\kappa + i\Delta + \omega_{im})t} \right] \\ &+ T_0 \sqrt{2\kappa_{ex}} a_{in} e^{-(\kappa + i\Delta + \omega_{im})(t + T_0)} \left[Ei\{(\kappa + i\Delta + \omega_{im}) T_0\} - Ei\{(\kappa + i\Delta + \omega_{im})(t + T_0)\} \right], \end{aligned} \tag{S.6}$$

where $Ei(t) = -\int_{-t}^{\infty} e^{-\tau}/\tau \, d\tau$ is the exponential integral. Therefore, the transmission coefficient is written as:

$$\tilde{T}(t) = 1 - \frac{2\kappa_{ex}}{\kappa + i\Delta + \omega_{im}}\left[1 - e^{-(\kappa + i\Delta + \omega_{im})t}\right]$$
$$+ 2\kappa_{ex} T_0 \, e^{-(\kappa + i\Delta + \omega_{im})(t + T_0)}\left[Ei\left\{(\kappa + i\Delta + \omega_{im})T_0\right\} - Ei\left\{(\kappa + i\Delta + \omega_{im})(t + T_0)\right\}\right]. \quad \text{(S.7)}$$

Although Eq. (S.7) is rather complicated, the transmission coefficient $\tilde{T}(t)$ eventually converges to its quasi-steady state $\tilde{T}_{qs} \equiv \frac{\kappa_i - \kappa_{ex} + i\Delta + \omega_{im}}{\kappa_i + \kappa_{ex} + i\Delta + \omega_{im}}$ in time. The smooth filter function describing the start of the excitation modifies the transient, but it does not affect the quasi-steady state transmission.

II. Numerical Analysis: temporal and frequency responses of systems under complex frequency excitations

One common method to obtain frequency responses of systems subject to continuous and monochromatic excitations is to apply a Fourier transform to their temporal response. It is important to note, however, that this approach can only be used for inputs which are finite in time, since sometimes complex frequency signals are not orthogonal and are unbounded. In particular, when impinging waves indefinitely grow (decay) in time after (before) t = 0, there are no Fourier pairs for the waves. Therefore, it is impossible to use Fourier transforms to calculate the frequency response for impinging waves that oscillate at a growing or decaying rate unless they stop at some point rather than diverging in time. In practice, these excitations are always finite in time, and imply that the excitation is non-monochromatic. As explained in Supplementary Section I, the transmission coefficient under complex frequency excitations can be divided into a quasi-steady and a transient state. The quasi-steady state transmission coefficient does not change over time,

since the system oscillates at the same complex frequency as the excitation, while the transient does change and it depends on the initial conditions and excitation. The transient portion of the transmission coefficient decays over time, and hence we can determine the quasi-steady state by waiting sufficiently long, if we can make sure that the excitation at a complex frequency is maintained past the transient. For instance, when input waves are growing in the black dotted curve of Fig. S1(a), we can obtain the steady state in Eq. (S.4) and rewrite the relation as:

$$\lim_{t\to\infty}\tilde{T}(t)=\tilde{T}_{qs}=\frac{\kappa_i-\kappa_{ex}+i\Delta+\omega_{im}}{\kappa_i+\kappa_{ex}+i\Delta+\omega_{im}}. \tag{S.8}$$

When the condition $\omega_{im}=\kappa_{ex}-\kappa_i$ in Eq. (2) of the main paper is met, the transmission in Eq. (S.8) reaches zero due to virtual critical coupling. Figure S1(b) depicts the temporal evolution of the intensity transmission $T(t)=\left|\tilde{T}(t)\right|^2$ at resonance for this optimal complex frequency excitation, demonstrating that the transmission approaches zero after a transient. Using Eq. (S.8), we can numerically calculate the frequency response of the transmission by sweeping the detuning Δ over the frequency range of interest, as shown in Fig. S1(c). We must note that the intensity transmission is identical to the transmissions used in the main text.

In practical applications, we are unable to use an infinitely growing incident wave and instead use a finite pulse, which must be released after growing according to the orange curve in Fig. S1(a). The temporal response of the transmission under the finite pulse excitation (orange curve) in Fig. S1(b) is similar to the response to the infinitely growing input (black dashed curve) up until $t\kappa_{ex}\approx 5$, where the finite pulse is released. Here, the resonator is highly overcoupled to a waveguide, i.e., $\kappa_{ex}/\kappa_i=4$. The blue-shaded area indicates the quasi-stationary regime, showing $\left|\tilde{T}(t)\right|^2\approx\left|\tilde{T}_{qs}\right|^2$. We determine the corresponding transmission spectrum with the finite pulse after collecting quasi-

steady states with varied detuning in Figure S1(c), and it is remarkably similar to the transmission spectrum with the infinitely growing input. This indicates that a realistic and finite pulse with a growing rate that satisfies the condition in Eq. (2) can provide virtual critical coupling once it enters the quasi-stationary regime.

We further investigate the impact of dynamically tuning the imaginary component of the complex frequency of impinging pulses ω_{im} on the system response. Specifically, we examine how the system response changes as the detuning is varied. In Fig. S1(b), the transmission spectra are presented with increasing ω_{im} from zero (blue) to $10\left(\kappa_{ex}-\kappa_i\right)$ (orange) for $\kappa_{ex}/\kappa_i=4$. Like above, the transmission spectra are obtained after the system reaches quasi-steady state. Our results demonstrate that the coupling regime between a resonator and a waveguide can be controlled freely by varying the growth rate of pulses.

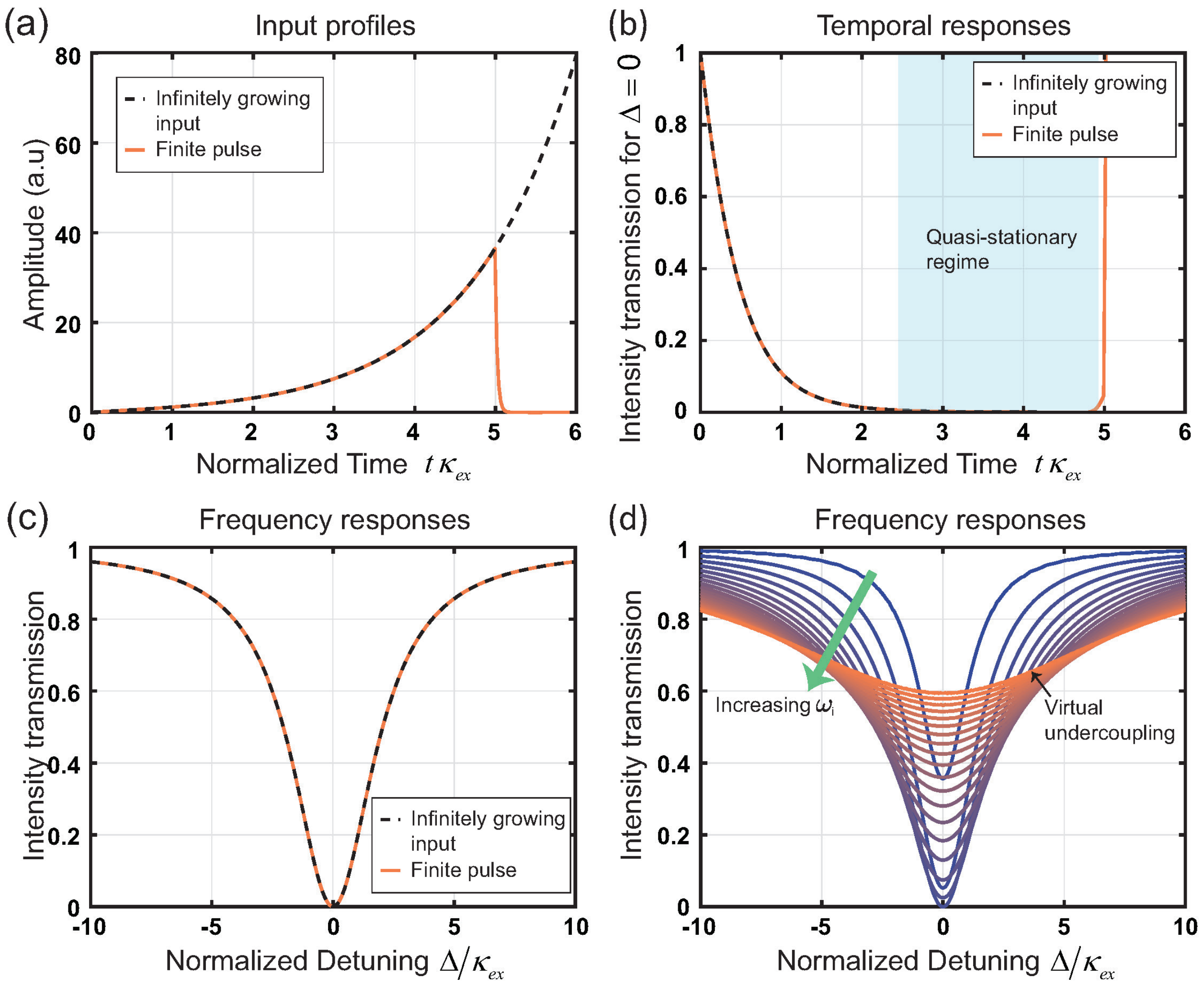

Figure S1 (a) Input profiles for infinitely growing input (black dotted) and finite pulse (orange). Both grow at the rate $\omega_{im} = \kappa_{ex} - \kappa_i$, and the finite pulse is released at $t\kappa_{ex} \approx 5$. (b) Temporal evolutions of the transmissions for an infinitely growing input and a finite pulse. The transmission is close to zero, showing the virtual critical coupling in the quasi-steady state under the finite pulse (blue-shaded area). (c) Frequency responses of the transmissions for both cases. The curves are identical to each other and are very close to the transmission spectrum of the critical coupling case in Fig. 1 of the main text. (d) Frequency responses of the transmissions while varying ω_i . Transmissions evolve from overcoupling to virtual undercoupling after passing through virtual critical coupling when the transmission dip is zero at the zero detuning.

III. Virtual critical coupling for an undercoupled resonator

In the previous sections, we discussed the virtual critical coupling obtained by an exponentially-growing impinging pulse in a highly overcoupled system. However, critical coupling can also be achieved in undercoupled systems with exponentially-decaying impinging pulses, and Eq. (S.4) remains applicable in this case. To observe virtual critical coupling, however, we must build up the impinging pulses before releasing them, as shown in Fig. S2(a) with different decay rates $\omega_{im}/\omega_{crit} = 0.5$, $\omega_{im}/\omega_{crit} = 1$, and $\omega_{im}/\omega_{crit} = 1.25$, where ω_{im} is the imaginary frequency of impinging waves and ω_{crit} is the critical coupling condition, i.e., $\omega_{crit} = \kappa_{ex} - \kappa_i$. In this case, we focus on the undercoupled system whose intrinsic loss is given by $\kappa_i/\kappa_{ex} = 4$. Similar to the previous case, we measure the temporal intensity transmissions for the three cases when the pump laser is at the resonance of the mode, as shown in Fig. S2(b). After $\tau\kappa_{ex} > 6$, all transmissions converge in time to the predicted values 0.18, 0, and 0.36, respectively for $\omega_{im}/\omega_{crit} = 0.5$, $\omega_{im}/\omega_{crit} = 1$, and $\omega_{im}/\omega_{crit} = 1.25$ in Eq. (S.8). Figure S2(c) illustrates the corresponding transmission spectrum by measuring each quasi-steady state for wide-ranging detuning. As expected, the transmission dip at $\Delta = 0$ is identical to the values for corresponding decaying rates ω_{im} in quasi-steady states. We also compare the frequency response for $\omega_{im}/\omega_{crit} = 1$ with transmissions for an undercoupled resonator (blue) and under the critical coupling condition (black dashed) in Fig. S2(d). Our results indicate that the realistic and finite decaying pulse can compensate for intrinsic loss inside an undercoupled resonator through virtual gain, enabling conjugate matching between a resonator and a waveguide [5].

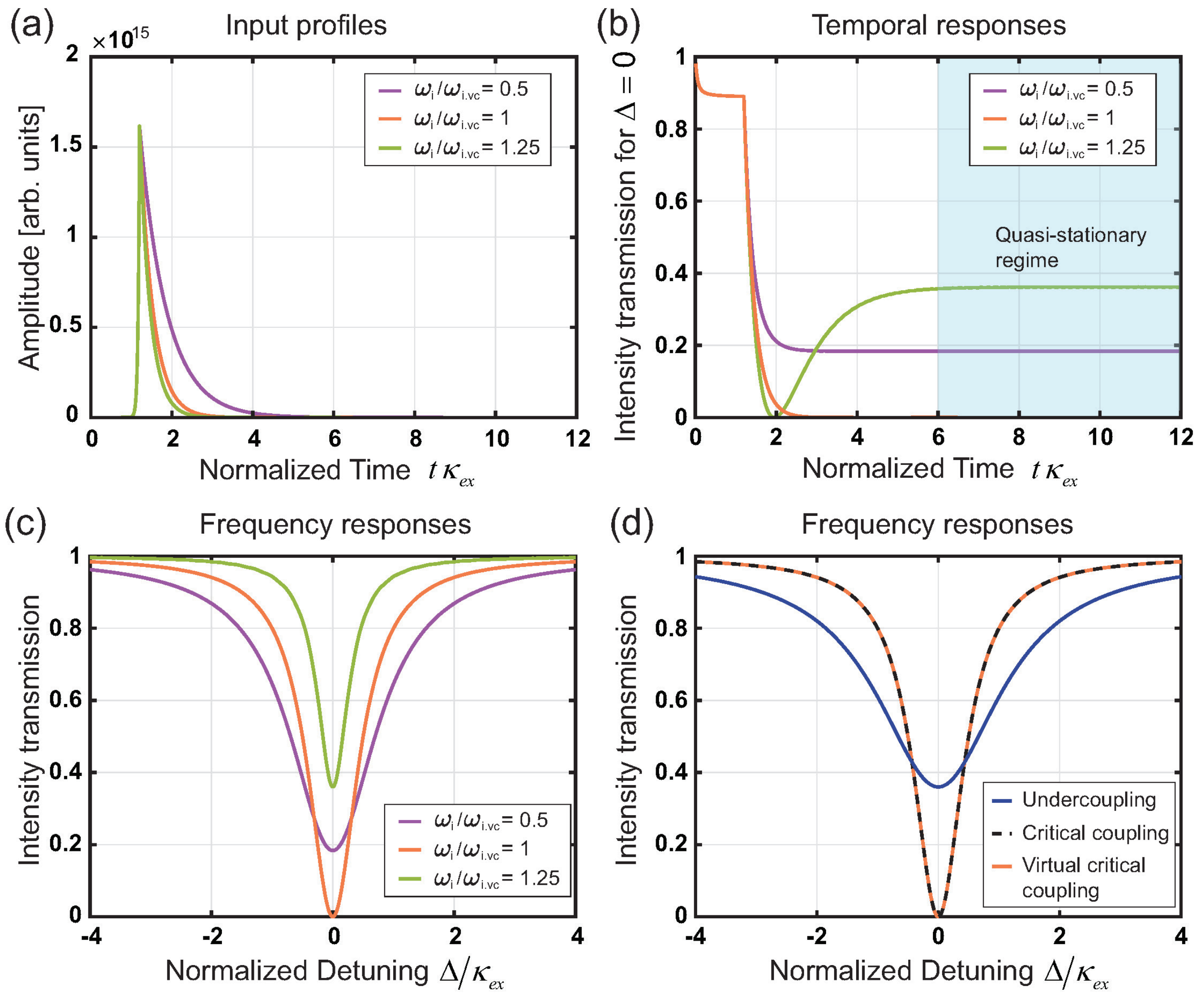

Figure S2 (a) Profiles of input pulses with different decay rates $\omega_{im}/\omega_{crit} = 0.5$ (green), $\omega_{im}/\omega_{crit} = 1$ (orange), and $\omega_{im}/\omega_{crit} = 1.25$ (purple). These three pulses are released at the same time after they grow at the same rate. (b) Temporal responses for the three different impinging pulses. The transmission for $\omega_{im}/\omega_{crit} = 1$ converges to zero after $t\kappa_{ex} > 2$, showing virtual critical coupling in the quasi-steady state. (c) Corresponding frequency responses for the three cases. At $\Delta = 0$, the transmission dips are identical to the values in the quasi-steady regime in Fig. S2(b). (d) Frequency responses under the virtual critical coupling (orange), critical coupling (black dashed), and undercoupling conditions (blue).

References

[1] H. Haus, *Waves and Fields in Optoelectronics*. Prentice-Hall, Englewood Cliffs, NJ, 1984.

[2] C. Gardiner, P. Zoller, and P. Zoller, *Quantum noise: a handbook of Markovian and non-Markovian quantum stochastic methods with applications to quantum optics*. Springer Science & Business Media, 2004.

[3] C. Manolatou, M. Khan, S. Fan, P. Villeneuve, H. Haus, and J. Joannopoulos, "Coupling of modes analysis of resonant channel add-drop filters," *IEEE Journal of Quantum Electronics*, vol. 35, no. 9, pp. 1322–1331, 1999.

[4] Y. Ra'di, A. Krasnok, and A. Alù, "Virtual critical coupling," *ACS Photonics*, vol. 7, no. 6, pp. 1468–1475, 2020.

[5] S. Kim, S. Lepeshov, A. Krasnok, and A. Alù, "Beyond bounds on light scattering with complex frequency excitations," *Phys. Rev. Lett.*, vol. 129, pp. 203601, 2022.
